# Impact of concomitant methotrexate on disease activity in patients with rheumatoid arthritis tapering abatacept: results from KOBIO registry

**DOI:** 10.3389/fmed.2024.1418243

**Published:** 2024-07-22

**Authors:** Jun Won Park, Ju Yeon Kim, Min Jung Kim, Yoo Kyoung Lim, Hyoun-Ah Kim, Jin Hyun Kim, Kichul Shin

**Affiliations:** ^1^Division of Rheumatology, Department of Internal Medicine, College of Medicine, Seoul National University, Seoul, Republic of Korea; ^2^Division of Rheumatology, Department of Internal Medicine, Seoul Metropolitan Government—Seoul Boramae Medical Center, Seoul, Republic of Korea; ^3^Department of Internal Medicine, Seoul Metropolitan Government—Seoul Boramae Medical Center, Seoul, Republic of Korea; ^4^Department of Rheumatology, Ajou University School of Medicine, Suwon, Republic of Korea; ^5^Division of Rheumatology, Department of Internal Medicine, Chungnam National University College of Medicine, Daejeon, Republic of Korea

**Keywords:** rheumatoid arthritis, abatacept, methotrexate, tapering, disease activity

## Abstract

**Objectives:**

Tapering biologic agents can be considered for patients with stable disease activity in rheumatoid arthritis (RA). However, the specific strategy for abatacept is uncertain. This study aimed to examine the impact of tapering abatacept on disease activity in RA patients and assess the potential influence of concomitant methotrexate (MTX) treatment.

**Methods:**

Using data from the KOBIO registry, we included 505 1 year intervals from 176 patients with RA that initiated abatacept with concomitant MTX at baseline. The intervals were divided into two groups based on the dose quotient (DQ) of abatacept during each period (i.e., the tapering group (DQ < 1) and control group (DQ = 1)). The primary outcome was achieving DAS28-remission at 1 year intervals. Marginal structural models (MSM) were used to minimize confounding caused by an imbalance in time-varying variables.

**Results:**

Abatacept was tapered at 146 (28.9%) intervals, and the mean DQ was 0.68. DAS28-remission was achieved in 207 (41.8%) intervals. Tapering abatacept did not affect the odds of achieving DAS28-remission compared with the control group (OR 1.04 [0.67–1.62]). The odds remained unaffected in the subgroup that continued MTX (OR 1.42 [0.88–2.30]) but not in the subgroup that discontinued MTX (OR 0.26 [0.10–0.57]). The effects of interaction between tapering abatacept and concomitant MTX use on DAS28 and patient’s functional status showed consistent results. The incidence of adverse events within a 1 year interval was comparable between the two groups.

**Conclusion:**

Withdrawal of MTX while tapering abatacept may compromise meeting the treatment goal for patients with RA.

## Introduction

Rheumatoid arthritis (RA) is a chronic autoimmune disease characterized by progressive joint damage and functional impairment. Although the use of conventional synthetic disease-modifying anti-rheumatic drugs (csDMARDs) markedly improves outcomes, a significant proportion of patients show an inadequate response to treatment (1). In the last few decades, biological DMARDs targeting various pathways involved in RA pathogenesis have been introduced, and their efficacy and safety have been demonstrated in many clinical trials and cohort studies (2, 3). However, long-term use of bDMARDs could increase the risk of adverse events such as infectious complications and could be a significant economic burden to patients and their healthcare system (4–7).

Several clinical studies have shown that tapering bDMARDs after achieving a treatment target has an efficacy comparable to standard-dose treatment (8–11). Based on these results, a recent EULAR guideline suggested that dose reduction of bDMARDs and/or csDMARDs may be a viable treatment option (12). However, previous studies on the tapering strategy mainly included patients treated with TNF inhibitors, and there needs to be more evidence regarding the efficacy of tapering non-TNFi bDMARDs (13). The lack of a standardized protocol for the tapering strategy is another hurdle for its implementation. One such example is concomitant methotrexate (MTX) treatment. Although the ACR and EULAR guidelines recommend the continuation of MTX during bDMARD treatment, many cohort studies have shown lower adherence to MTX in patients with bDMARDs (12, 14–17). However, no studies have investigated the effect of tapering or withdrawal of MTX in patients who underwent tapering of their bDMARD treatment.

Abatacept is a selective modulator of the CD80/86:CD28 co-stimulatory signal for T cell activation. While its long-term efficacy and safety have been established, only a few studies investigated the effect of tapering abatacept on disease activity in patients with RA (18–20). In this study, we aimed to examine the impact of tapering abatacept on disease activity in RA patients and assess the potential influence of concomitant MTX treatment.

## Methods

### Study population

All data were collected from the Korean College of Rheumatology Biologics and Targeted Therapy Registry (KOBIO-RA), a nationwide longitudinal cohort of RA patients receiving bDMARD at 47 referral medical centers in South Korea (NCT01965132) (21). By December 2021, 2,701 patients were enrolled and followed up annually. A patient was included in the registry when starting a new bDMARD (baseline visit) and was followed up annually. If the patient discontinued the bDMARD, clinical data, including disease activity and reason for its discontinuation, were collected.

In the KOBIO-RA registry, we included patients with available follow-up data who received MTX concomitantly at the baseline visit and continued it for at least 1 year. Patients who 1) received a tapered dose of abatacept at baseline, 2) withdrew consent, or 3) had no follow-up data available were excluded (Supplementary Figure S1).

This study was conducted following the Declaration of Helsinki. It was approved by the Institutional Review Board (IRB) of the coordinating center (Seoul Metropolitan Government, Seoul Boramae Medical Centre, Seoul, Korea [IRB No. 26-2012-34]) and by each participating referral center. Written informed consent was obtained from all patients.

### Data collection

Data on demographics, body mass index (BMI), smoking status (never, ex-smoker, or current smoker), comorbidities, and previous bDMARD treatment were collected at baseline. For the assessment of disease activity, we collected data on swollen/tender joint count (0–44), patient and physician global assessment (0–10), erythrocyte sedimentation rate (ESR) level, and C-reactive protein (CRP) level at baseline and annual follow-up visits to calculate the Disease Activity Score-28 with ESR (DAS28-ESR) and Simplified Disease Activity Index (SDAI). The functional status of the patients was estimated using the Health Assessment Questionnaire Disability Index (HAQ-DI), which was converted from the Multidimensional Health Assessment Questionnaire (MDHAQ) using a validated formula (22). Observation of each patient was terminated at 1) discontinuation of abatacept for any cause, 2) the last follow-up visit, or 3) December 31, 2021, whichever came first.

The abatacept dose and interval were recorded at each follow-up visit. We estimated the quantity of tapering abatacept using the dose quotient (DQ), calculated as (mean actual dose/standard dose) × (standard − dosing interval/mean actual dosing interval) (23). The tapering of abatacept at each follow-up visit was determined based on the decrease in the DQ compared with the previous visit. All 1 year intervals were classified into tapering and control groups using this criterion. Data on concomitant medications, such as MTX, and their dosage were collected every visit.

### Outcomes

The primary outcome of this study was remission based on DAS28-ESR (<2.6) at each follow-up visit for all included patients. Secondary outcomes included achieving SDAI remission (≤3.3) and DAS28-low disease activity (LDA) (<3.2). Disease activity measured using the DAS28-ESR, SDAI, and HAQ-DI was also included as a secondary outcome. Severe functional impairment was defined as a HAQ-DI score >2.0. In addition, the proportion of 1 year intervals in which adverse events (AEs) occurred between the two groups was examined. Serious adverse events (SAEs) were defined as events that resulted in marked limitations in activity, required medical intervention or therapy, or led to hospitalization for AE management.

### Statistical analysis

In our database, the proportion of missing values in the clinical characteristics was less than 5%; therefore, no imputation was performed. In addition, we confirmed that the distribution of the missing values was completely at random by Little’s missing completely at random (MCAR) test (*p* = 0.157). Since the tapering of abatacept and disease activity could be influenced by several time-varying confounders, such as disease activity and prior dose of abatacept (Supplementary Figure S2), we used marginal structural models (MSM) to obtain unbiased estimates by considering the effect of time-varying confounders (24). Briefly, the inverse probability of treatment weighting (IPTW) was calculated. Treatment weights for each follow-up visit were calculated based on the likelihood of tapering abatacept. All clinical factors measured at baseline and immediately before the visit were included in the prediction model as covariates. We trimmed the upper and lower 1% percentiles because observations with extreme weight could have biased the results. To address the attrition bias due to loss to follow-up, we calculated the censoring weights for an unbiased study sample. The final weights are obtained by multiplying the two weights. The covariate balance was assessed using the standardized mean differences (SMD). An SMD of <0.1 indicates a lack of imbalance between variables.

In the second stage, the effect of tapering bDMARD on the outcome was assessed in the pseudo-population using a generalized estimating equation (GEE) to consider repeated measurements for each patient. An “Autoregressive-1” correlation matrix was used based on the correlation coefficients among the best-fit data. When the effect of tapering abatacept was assessed after stratification according to the presence of clinical factors, the subgroup-specific IPTW was calculated and applied to the analysis. The safety assessment of abatacept in the two groups was compared without applying IPTW. All statistical analyses were performed using R (4.2.1), and *p* values less than 0.05 were considered statistically significant.

## Results

### Baseline characteristics

In total, 179 patients were included in this study. The baseline characteristics of the patients are presented in Supplementary Table S1. The mean (SD) age of the included patients was 59.3 (12.9), and 83.8% (*n* = 150) were female. One-hundred sixty-three patients were seropositive for RA, and 134 (87.0%) were anti-citrullinated peptide antibodies (ACPA)-positive. The mean disease duration of RA was 8.9 (3.3) years, and most patients (*n* = 141, 78.8%) had not been treated with other targeted agents before the abatacept treatment. Twenty-three patients (12.8%) received subcutaneous agent. The mean DAS28-ESR and HAQ were 5.66 (0.99) and 1.11 (0.68), respectively. The mean weekly dose of MTX at baseline was 12.5 (3.3) mg. A total of 149 patients (83.2%) used glucocorticoid at the baseline with a mean (SD) dose of 4.8 (3.9) mg/day of prednisolone.

### Tapering of abatacept during the follow-up

A total of 505 1 year intervals were analyzed from the included patients, with an observation period of 497.5 person-years. During the observation period, abatacept was tapered at 146 (28.9%) intervals, with a mean (SD) DQ of 0.68 (0.14). In the tapering group, DAS28-ESR before the tapering was 3.37 (1.35); 51 (34.9%), and 77 (52.7%) achieved remission and low disease activity, respectively. The proportion of 1 year intervals achieving DAS28-remission or low disease activity was numerically higher in the tapering group, compared with that in the control group (44.3% vs. 52.7%, *p* = 0.170). In contrast, the mean HAQ-DI before tapering was 0.80 (0.57), and only nine (6.1%) patients showed severe functional impairment before tapering abatacept. Of the total intervals, 91 (18.0%) involved discontinuation of MTX. Among these intervals, 32 (35.1%) were accompanied by concomitant tapering of abatacept.

There were significant differences in the disease activity and concomitant medications at the previous visit between the control and tapering groups. In the tapering group, patients showed lower disease activity, less frequent concomitant glucocorticoid treatment, and less frequent tapering of abatacept during the prior visit than those in the control group. All covariates were well-balanced after applying IPTW (Table 1).

**Table 1:** Clinical features of the subjects included at 1 year intervals.

	Control group (*n* = 359)	Tapering group (*n* = 146)	SMD before IPTW	SMD after IPTW
Age, years, mean (SD)	57.1 (13.4)	59.4 (12.3)	0.180	0.078
Female sex, *n* (%)	313 (87.2)	124 (84.9)	0.065	0.020
BMI, mean (SD)	21.9 (3.1)	22.4 (3.1)	0.150	0.089
Disease duration, years, mean (SD)	9.6 (8.4)	9.5 (8.5)	0.010	0.010
Smoking, *n* (%)			0.123	0.020
Never	302 (84.1)	127 (87.0)		
Ex	26 (7.2)	11 (7.5)		
Current	31 (8.6)	8 (5.5)		
Interstitial lung disease, *n* (%)	18 (5.0)	4 (2.7)	0.118	0.025
Hypertension, *n* (%)	117 (32.6)	45 (30.8)	0.038	0.022
Diabetes, *n* (%)	32 (8.9)	13 (8.9)	<0.001	0.060
Dyslipidemia, *n* (%)	65 (18.1)	23 (15.8)	0.063	0.056
Osteoporosis, *n* (%)	127 (35.4)	48 (32.9)	0.053	0.005
Hypothyroidism, *n* (%)	21 (5.8)	12 (8.2)	0.093	0.062
Seropositive RA, *n* (%)	327 (91.1)	138 (94.5)	0.133	0.033
Erosion on radiographs, *n* (%)	181 (50.4)	61 (41.8)	0.174	0.066
bDMARD/tsDMARD-naïve, *n* (%)	271 (75.5)	114 (78.1)	0.061	0.006
Baseline DAS28-ESR, mean (SD)	5.68 (1.00)	5.60 (0.95)	0.082	0.009
Baseline HAQ, mean (SD)	1.13 (0.69)	1.06 (0.68)	0.098	0.019
Baseline MTX dose, mg, mean (SD)	12.7 (3.1)	12.4 (3.0)	0.101	0.015
Baseline GC use, n (%)	295 (82.2)	115 (78.8)	0.086	0.048
Baseline GC dose^a^, mg, mean (SD)	5.1 (4.5)	4.4 (3.9)	0.169	0.022
DAS28-ESR in the previous interval, mean (SD)	3.64 (1.40)	3.37 (1.35)	0.197	0.020
HAQ in the previous interval, mean (SD)	0.90 (0.65)	0.80 (0.57)	0.168	0.002
MTX use in the previous interval, *n* (%)	322 (89.7)	125 (85.6)	0.124	0.044
GC use in the previous interval, *n* (%)	259 (72.1)	101 (69.2)	0.065	0.036
GC dose in the previous interval, mg, mean (SD)^a^	3.5 (3.5)	3.0 (3.0)	0.135	0.053
Tapered dose of abatacept in the previous interval, *n* (%)	127 (35.4)	30 (20.5)	0.335	0.029

BMI, body mass index; CI, confidence interval; DAS, disease activity score; ESR, erythrocyte sedimentation rate; GC, glucocorticoid; HAQ, health assessment questionnaire; IPTW, inverse probability of treatment weighting; MTX, methotrexate; SMD, standardized mean difference.

a Based on dose of prednisolone.

### Effect of tapering abatacept on achieving DAS28-remission

**Figure 1:**
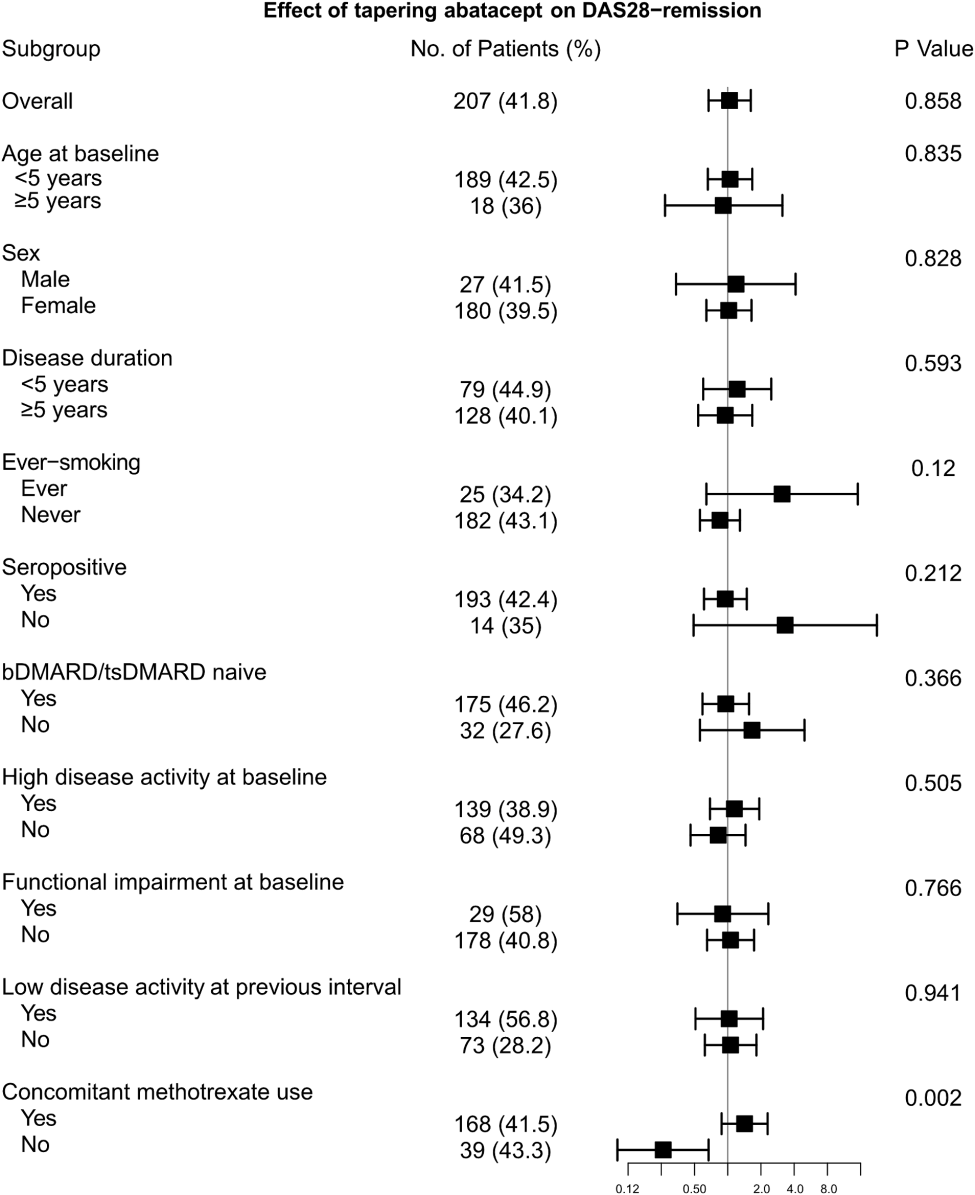
Effect of tapering abatacept on the likelihood of achieving DAS28-remission stratified by clinical features.

Remission based on DAS28-ESR was achieved at 207 (41.8%) intervals, and its proportion was comparable between the control and tapering groups (40.5% vs. 45.1%, *p* = 0.337). In the IPTW-applied pseudopopulation, tapering of abatacept also did not significantly affect the odds of achieving DAS28-remission (OR 1.04 [95% CI 0.67 to 1.62]). However, the impact of tapering differed substantially according to concomitant MTX use (Figure 1). In the subgroup of intervals without MTX, tapering abatacept significantly decreased the possibility of achieving the target (OR 0.26 [0.10 to 0.57]). By contrast, with concomitant MTX therapy, the effect of tapering was not significant (OR 1.42 [0.88 to 2.30]). This result was consistent when the tapering effect was estimated in the pseudopopulation composed of 1 year intervals not treated with concomitant MTX (OR 0.33 [0.11 to 0.98]). In addition, the impact of tapering abatacept did not change in conventional GEE analysis after adjusting for baseline and time-varying factors (Supplementary Table S2). Furthermore, to estimate the effect of tapering abatacept in conjunction with concomitant MTX treatment, we performed another GEE analysis which included DQ multiplied by 100 in the model instead of using a categorical variable for tapering abatacept. This analysis showed that tapering the dose of abatacept significantly decreased the likelihood of achieving DAS28-remission with dose-dependent manner (adjusted OR 0.96 [0.94 to 0.99]) (Supplementary Table S3).

Contrary to the concomitant use of MTX, the effect of tapering was consistent when the primary analysis was stratified according to age, sex, smoking status, disease duration, previous bDMARD treatment, and baseline disease activity (Figure 1). Notably, achieving DAS28-LDA in the interval before tapering abatacept also had no significant effect on disease activity after the tapering abatacept.

### Effect of tapering abatacept on other efficacy outcomes

In the IPTW-applied pseudopopulation, tapering abatacept also significantly reduced the likelihood of achieving DAS28-LDA only in intervals without concomitant MTX. The consistency of the results was reaffirmed in the subsequent analysis in which the DAS28-ESR score served as an outcome variable (Table 2). Contrary to DAS28-remission, stricter remission criteria based on the SDAI were achieved in only 79 (16.0%) intervals. Tapering abatacept was not associated with the likelihood of attaining SDAI remission; however, the effect did not change with concomitant MTX.

**Table 2:** Effect of tapering abatacept on other disease activity indices based on concomitant MTX use.

	OR (95% CI)	*p*	*p* for interaction
DAS28-LDA			0.003
With concomitant MTX	1.06 (0.75 to 1.50)	0.734	
Without concomitant MTX	0.20 (0.07 to 0.55)	0.002	
SDAI-remission			0.446
With concomitant MTX	0.85 (0.46 to 1.57)	0.597	
Without concomitant MTX	0.50 (0.15 to 1.62)	0.247	
SDAI-LDA			0.179
With concomitant MTX	1.06 (0.71 to 1.57)	0.781	
Without concomitant MTX	0.47 (0.16 to 1.37)	0.167	
Functional impairment^a^			0.045
With concomitant MTX	0.56 (0.18 to 1.80)	0.334	
Without concomitant MTX	4.27 (0.86 to 21.32)	0.077	

	*β* (95% CI)	*p*	*p* for interaction
DAS28-ESR score			0.002
With concomitant MTX	−0.02 (−0.19 to 0.16)	0.849	
Without concomitant MTX	0.79 (0.32 to 1.26)	0.001	
SDAI score			0.120
With concomitant MTX	−0.60 (−1.80 to 0.60)	0.330	
Without concomitant MTX	2.58 (−1.20 to 6.35)	0.180	
HAQ			0.101
With concomitant MTX	0.01 (−0.09 to 0.11)	0.820	
Without concomitant MTX	0.20 (0.007 to 0.39)	0.042	

CI, confidence interval; DAS, disease activity score; ESR, erythrocyte sedimentation rate; HAQ, health assessment questionnaire; LDA, low disease activity; MTX, methotrexate; OR, odds ratio.

a Defined as HAQ > 2.0.

Tapering abatacept without concomitant MTX significantly reduced the functional status measured by HAQ (*β* = 0.20 [95% CI 0.01 to 0.39]) and tended to increase the likelihood of experiencing a severe functional impairment (OR 4.27 [0.86 to 21.32]). However, tapering the abatacept dose did not influence the HAQ when the patient concomitantly received MTX.

### Safety of tapering abatacept

During the observation period, 53 adverse events occurred in 46 (8.9%) intervals. The most common AEs were leukopenia (*n* = 10), followed by interstitial lung disease (*n* = 9), and herpes zoster infection (*n* = 7). No tuberculosis infections occurred during the observation period. There were no significant differences in the rates of AEs between the two groups (Table 3). Concomitant MTX use did not significantly affect the risk of occurrence of any AEs (OR 0.77 [0.36 to 1.66]) (Supplementary Table S3). Most AEs during the observation period were mild to moderate in severity, and only three SAEs occurred in the study population.

**Table 3:** Adverse events during the observation period.

	Control group (*n* = 359)	Tapering group (*n* = 146)	*p*
Any AEs^a^	31 (8.6)	13 (8.9)	0.923
Leukopenia	7 (1.9)	4 (2.7)	0.581
Infusion/injection site reaction	4 (1.1)	0 (0.0)	0.200
Tuberculosis infection	0 (0.0)	0 (0.0)	NA
NTM infection	1 (0.3)	0 (0.0)	0.523
Bacterial infection	4 (1.1)	2 (1.4)	0.810
Fungal infection	1 (0.3)	0 (0.0)	0.523
Herpes zoster infection	2 (0.6)	5 (3.4)	0.012
LFT abnormality	2 (0.6)	0 (0.0)	0.356
Skin rash	6 (1.7)	1 (0.7)	0.390
Interstitial lung disease	6 (1.7)	1 (0.7)	0.390
Malignancy	4 (1.1)	1 (0.7)	0.659
Any SAEs^b^	9 (2.5)	2 (1.4)	0.865
Bacterial infection	3 (0.8)	1 (0.7)	0.427
Fungal infection	1 (0.3)	0 (0.0)	0.523
Interstitial lung disease	2 (0.6)	0 (0.0)	0.366
Malignancy	4 (1.1)	1 (0.7)	0.659

AE, adverse event; LFT, liver function test; NTM, nontuberculous mycobacteria; NA, not applicable; SAE, serious adverse event.

a 37 and 14 AEs occurred in the control and the tapering groups, respectively.

b 10 and 2 SAEs occurred in the control and the tapering groups, respectively.

## Discussion

Although recent studies have suggested that tapering bDMARD could be a feasible option, the optimal tapering protocol to minimize the risk of flares remains uncertain, which is a significant hurdle for its implementation in daily clinical practice (13). One uncertainty in the tapering strategy is whether concomitant csDMARDs (mainly MTX) should be maintained during bDMARD tapering. Although a recent randomized controlled trial showed that tapering csDMARDs or TNFi had a similar effect on disease activity, this study did not include patients with concurrent tapering of both agents (25). To the best of our knowledge, this is the first nationwide study to investigate the impact of tapering abatacept dose in real-world patients with RA, focusing on the potential influence of concomitant MTX on its effectiveness.

In this study, we showed that the effect of tapering on the likelihood of achieving DAS28-remission differed significantly according to concomitant MTX use. At 1 year intervals with MTX, tapering abatacept did not significantly affect the likelihood of achieving DAS28-remission, which is consistent with previous studies that investigated the efficacy of tapering TNFi (8, 11). However, tapering abatacept dose and concurrent discontinuation of MTX were significantly associated with a reduced likelihood of achieving remission and an increased risk of functional impairment. This result is clinically significant because many real-world studies have shown a substantial decrease in MTX adherence after the initiation of bDMARD treatment (16, 26). Interestingly, a recent meta-analysis investigating the effect of tapering MTX in patients receiving combination treatment of MTX and various targeted agents demonstrated a 10% reduction in the likelihood of maintaining sustained remission upon tapering or withdrawal of MTX from targeted therapy (27). However, in this study, the combination of MTX discontinuation and tapering abatacept was associated with an approximately 70% reduction in the possibility of attaining DAS28-remission. These results suggest a compelling complementary effect of MTX and abatacept in controlling disease activity in patients with RA.

Our data also showed that the incidence of adverse events was comparable between the control and tapering groups. This was partially attributable to the low absolute incidence rate of each adverse event (less than 5%). Notably, concomitant MTX use did not significantly increase the risk of AEs, again emphasizing that the potential benefit of MTX treatment outweighs the potential risk of AEs.

Recent EULAR guidelines on tapering bDMARD recommend that tapering be considered after achieving sustained remission. However, many cohort studies have demonstrated that patients in the real world rarely reach this target, especially those with established RA (28–30). We showed that tapering abatacept was performed at approximately 30% of the 1 year intervals, suggesting that the tapering strategy is a relatively common practice in South Korea. However, the proportion of intervals achieving the DAS28-remission and LDA before tapering abatacept was only 28.9 and 47.1%, respectively. This suggests a notable disparity in the indication for tapering abatacept between the current guidelines and real-world clinical practice. This discrepancy can be partially attributed to various factors that affect the patients’ treatment trajectory beyond disease activity. These factors include patient and physician preferences, comorbidities, risk of adverse events, and the cost of medications. We showed that the likelihood of achieving DAS28-remission/LDA was comparable between the control and tapering groups, irrespective of disease activity measured at the previous interval. These results suggest that tapering abatacept may be feasible when applied to more lenient indications in real-world clinical settings. Our results should be confirmed in future large-scale studies to appropriately implement this strategy in the real world.

This study has several limitations. First, since this study was observational, the comparative efficacy of the tapering group can be biased by confounding by indication. Although we used MSM to minimize them, some unmeasured confounders could affect the decision to taper abatacept and potentially bias the results. For example, the dose of abatacept could be influenced on patient’s compliance to medication and this unmeasured confounding factor could potentially bias the effect of tapering strategy. Therefore, our results should be validated in future randomized controlled studies. Second, the effect of abatacept and/or MTX tapering on the radiographic progression of RA was not assessed. Finally, because the KOBIO cohort collects follow-up data annually, it is possible that changes in disease activity and treatment adjustment could not be captured.

In conclusion, our study indicates that tapering abatacept is a viable option for patients with stable RA when concurrently treated with MTX. However, the effectiveness of abatacept after its tapering can be compromised upon withdrawal of concomitant MTX.

## Data availability statement

The data underlying this study are available from the corresponding authors upon reasonable request.

## Ethics statement

The studies involving humans were approved by Institutional Review Board (IRB) of Seoul Metropolitan Government, Seoul Boramae Medical Centre. The studies were conducted in accordance with the local legislation and institutional requirements. The participants provided their written informed consent to participate in this study.

## Author contributions

JP: Conceptualization, Data curation, Formal analysis, Investigation, Methodology, Writing – original draft, Writing – review & editing. JuK: Writing – review & editing. MK: Writing – review & editing. YL: Writing – review & editing. H-AK: Writing – review & editing. JiK: Writing – review & editing. KS: Conceptualization, Data curation, Investigation, Supervision, Writing – review & editing.

## References

[1] Smolen JS Aletaha D McInnes IB. Rheumatoid arthritis. Lancet. (2016) 388:2023–38. doi: 10.1016/S0140-6736(16)30173-8, PMID: 27156434

[2] Haugeberg G Hansen IJ Soldal DM Sokka T. Ten years of change in clinical disease status and treatment in rheumatoid arthritis: results based on standardized monitoring of patients in an ordinary outpatient clinic in southern Norway. Arthritis Res Ther. (2015) 17:219. doi: 10.1186/s13075-015-0716-0, PMID: 26290061 PMC4545980

[3] Nam JL Takase-Minegishi K Ramiro S Chatzidionysiou K Smolen JS van der Heijde D. Efficacy of biological disease-modifying antirheumatic drugs: a systematic literature review informing the 2016 update of the EULAR recommendations for the management of rheumatoid arthritis. Ann Rheum Dis. (2017) 76:1113–36. doi: 10.1136/annrheumdis-2016-210713, PMID: 28283512

[4] Joensuu JT Huoponen S Aaltonen KJ Konttinen YT Nordstrom D Blom M. The cost-effectiveness of biologics for the treatment of rheumatoid arthritis: a systematic review. PLoS One. (2015) 10:e0119683. doi: 10.1371/journal.pone.0119683, PMID: 25781999 PMC4363598

[5] Machado-Alba JE Machado-Duque ME Gaviria-Mendoza A Reyes JM Gamboa NC. Use of healthcare resources in a cohort of rheumatoid arthritis patients treated with biological disease-modifying antirheumatic drugs or tofacitinib. Clin Rheumatol. (2021) 40:1273–81. doi: 10.1007/s10067-020-05432-6, PMID: 32997316 PMC7943490

[6] Zhou VY Lacaille D Lu N Kopec JA Qian Y Nosyk B. Risk of severe infections after the introduction of biologic DMARDs in people with newly diagnosed rheumatoid arthritis: a population-based interrupted time-series analysis. Rheumatology (Oxford). (2023) 62:3858–65. doi: 10.1093/rheumatology/kead158 37014364 PMC10691931

[7] Bongartz T Sutton AJ Sweeting MJ Buchan I Matteson EL Montori V. Anti-TNF antibody therapy in rheumatoid arthritis and the risk of serious infections and malignancies: systematic review and meta-analysis of rare harmful effects in randomized controlled trials. JAMA. (2006) 295:2275–85. doi: 10.1001/jama.295.19.2275, PMID: 16705109

[8] Smolen JS Nash P Durez P Hall S Ilivanova E Irazoque-Palazuelos F. Maintenance, reduction, or withdrawal of etanercept after treatment with etanercept and methotrexate in patients with moderate rheumatoid arthritis (PRESERVE): a randomised controlled trial. Lancet. (2013) 381:918–29. doi: 10.1016/S0140-6736(12)61811-X 23332236

[9] van Herwaarden N van der Maas A Minten MJ van den Hoogen FH Kievit W van Vollenhoven RF. Disease activity guided dose reduction and withdrawal of adalimumab or etanercept compared with usual care in rheumatoid arthritis: open label, randomised controlled, non-inferiority trial. BMJ. (2015) 350:h1389. doi: 10.1136/bmj.h1389, PMID: 25858265 PMC4391970

[10] Emery P Hammoudeh M FitzGerald O Combe B Martin-Mola E Buch MH. Sustained remission with etanercept tapering in early rheumatoid arthritis. N Engl J Med. (2014) 371:1781–92. doi: 10.1056/NEJMoa1316133, PMID: 25372086

[11] van Vollenhoven RF Ostergaard M Leirisalo-Repo M Uhlig T Jansson M Larsson E. Full dose, reduced dose or discontinuation of etanercept in rheumatoid arthritis. Ann Rheum Dis. (2016) 75:52–8. doi: 10.1136/annrheumdis-2014-205726, PMID: 25873634 PMC4717401

[12] Smolen JS Landewe RBM Bergstra SA Kerschbaumer A Sepriano A Aletaha D. EULAR recommendations for the management of rheumatoid arthritis with synthetic and biological disease-modifying antirheumatic drugs: 2022 update. Ann Rheum Dis. (2023) 82:3–18. doi: 10.1136/ard-2022-223356, PMID: 36357155

[13] Schett G Emery P Tanaka Y Burmester G Pisetsky DS Naredo E. Tapering biologic and conventional DMARD therapy in rheumatoid arthritis: current evidence and future directions. Ann Rheum Dis. (2016) 75:1428–37. doi: 10.1136/annrheumdis-2016-209201, PMID: 27261493

[14] Fraenkel L Bathon JM England BR St Clair EW Arayssi T Carandang K. 2021 American College of Rheumatology Guideline for the treatment of rheumatoid arthritis. Arthritis Care Res. (2021) 73:924–39. doi: 10.1002/acr.24596, PMID: 34101387 PMC9273041

[15] Bouman CAM Tweehuysen L Haverkort D van den Ende CH van der Maas A den Broeder AA. Abatacept and tocilizumab tapering in rheumatoid arthritis patients: results of SONATA-a retrospective, exploratory cohort study. Rheumatol Adv Pract. (2018) 2:e8. doi: 10.1093/rap/rky008 PMC6649917 31431957

[16] Shimizu Y Tanaka E Inoue E Shidara K Sugimoto N Seto Y. Reduction of methotrexate and glucocorticoids use after the introduction of biological disease-modifying anti-rheumatic drugs in patients with rheumatoid arthritis in daily practice based on the IORRA cohort. Mod Rheumatol. (2018) 28:461–7. doi: 10.1080/14397595.2017.1369926, PMID: 28880684

[17] Ebina K Hashimoto M Yamamoto W Hirano T Hara R Katayama M. Drug tolerability and reasons for discontinuation of seven biologics in elderly patients with rheumatoid arthritis-the ANSWER cohort study. PLoS One. (2019) 14:e0216624. doi: 10.1371/journal.pone.0216624, PMID: 31067271 PMC6505948

[18] Blair HA Deeks ED. Abatacept: a review in rheumatoid arthritis. Drugs. (2017) 77:1221–33. doi: 10.1007/s40265-017-0775-4 28608166

[19] Yoshida S Kotani T Kimura Y Matsumura Y Yoshikawa A Tokai N. Efficacy of abatacept tapering therapy for sustained remission in patients with rheumatoid arthritis: prospective single-center study. Int J Rheum Dis. (2019) 22:81–9. doi: 10.1111/1756-185X.13384, PMID: 30168272

[20] Emery P Tanaka Y Bykerk VP Huizinga TWJ Citera G Bingham CO. Sustained remission and outcomes with abatacept plus methotrexate following stepwise dose De-escalation in patients with early rheumatoid arthritis. Rheumatol Ther. (2023) 10:707–27. doi: 10.1007/s40744-022-00519-9, PMID: 36869251 PMC10140217

[21] Kim J Koh JH Choi SJ Jeon CH Kwok SK Kim SK. KOBIO, the first web-based Korean biologics registry operated with a unified platform among distinct disease entities. J Rheum Dis. (2021) 28:176–82. doi: 10.4078/jrd.2021.28.4.176, PMID: 37476366 PMC10324910

[22] Anderson J Sayles H Curtis JR Wolfe F Michaud K. Converting modified health assessment questionnaire (HAQ), multidimensional HAQ, and HAQII scores into original HAQ scores using models developed with a large cohort of rheumatoid arthritis patients. Arthritis Care Res (Hoboken). (2010) 62:1481–8. doi: 10.1002/acr.20265, PMID: 20496428

[23] Park JW Kim HA Shin K Park YB Kim TH Song YW. Effects of tapering tumor necrosis factor inhibitor on the achievement of inactive disease in patients with axial spondyloarthritis: a nationwide cohort study. Arthritis Res Ther. (2019) 21:163. doi: 10.1186/s13075-019-1943-6, PMID: 31272498 PMC6611048

[24] Robins JM Hernan MA Brumback B. Marginal structural models and causal inference in epidemiology. Epidemiology. (2000) 11:550–60. doi: 10.1097/00001648-200009000-00011 10955408

[25] van Mulligen E de Jong PHP Kuijper TM van der Ven M Appels C Bijkerk C. Gradual tapering TNF inhibitors versus conventional synthetic DMARDs after achieving controlled disease in patients with rheumatoid arthritis: first-year results of the randomised controlled TARA study. Ann Rheum Dis. (2019) 78:746–53. doi: 10.1136/annrheumdis-2018-214970, PMID: 30954969

[26] Aaltonen KJ Turunen JH Sokka T Puolakka K Valleala H. A survey on the medication adherence to methotrexate among rheumatoid arthritis patients treated with self-administered biologic drugs. Clin Exp Rheumatol. (2016) 34:694–7. PMID: 27213997

[27] Meng CF Rajesh DA Jannat-Khah DP Jivanelli B Bykerk VP. Can patients with controlled rheumatoid arthritis taper methotrexate from targeted therapy and sustain remission? A systematic review and metaanalysis. J Rheumatol. (2023) 50:36–47. doi: 10.3899/jrheum.220152, PMID: 35970524

[28] Thomas K Lazarini A Kaltsonoudis E Drosos A Papalopoulos I Sidiropoulos P. Treatment patterns and achievement of the treat-to-target goals in a real-life rheumatoid arthritis patient cohort: data from 1317 patients. Ther Adv Musculoskelet Dis. (2020) 12:1759720X2093713. doi: 10.1177/1759720X20937132 PMC7534096 33062066

[29] PDH H Pauling JD McHugh N Shaddick G Hyrich K Group B-RC. Predictors, demographics and frequency of sustained remission and low disease activity in anti-tumour necrosis factor-treated rheumatoid arthritis patients. Rheumatology. (2019) 58:2162–9. doi: 10.1093/rheumatology/kez188 31155669 PMC6880851

[30] Einarsson JT Willim M Ernestam S Saxne T Geborek P Kapetanovic MC. Prevalence of sustained remission in rheumatoid arthritis: impact of criteria sets and disease duration, a Nationwide study in Sweden. Rheumatology. (2019) 58:227–36. doi: 10.1093/rheumatology/key054, PMID: 29538755

