# Supplementary material for: Impact of concomitant methotrexate on disease activity in patients with rheumatoid arthritis tapering abatacept: results from KOBIO registry
Source: Front Med (Lausanne). 2024 Jul 22;11:1418243. doi: 10.3389/fmed.2024.1418243 (PMC11298490; doi:10.3389/fmed.2024.1418243)
Supplement: Supplementary file 1 [file Data_Sheet_1.docx]

Supplementary Figure 1. Flow of this study


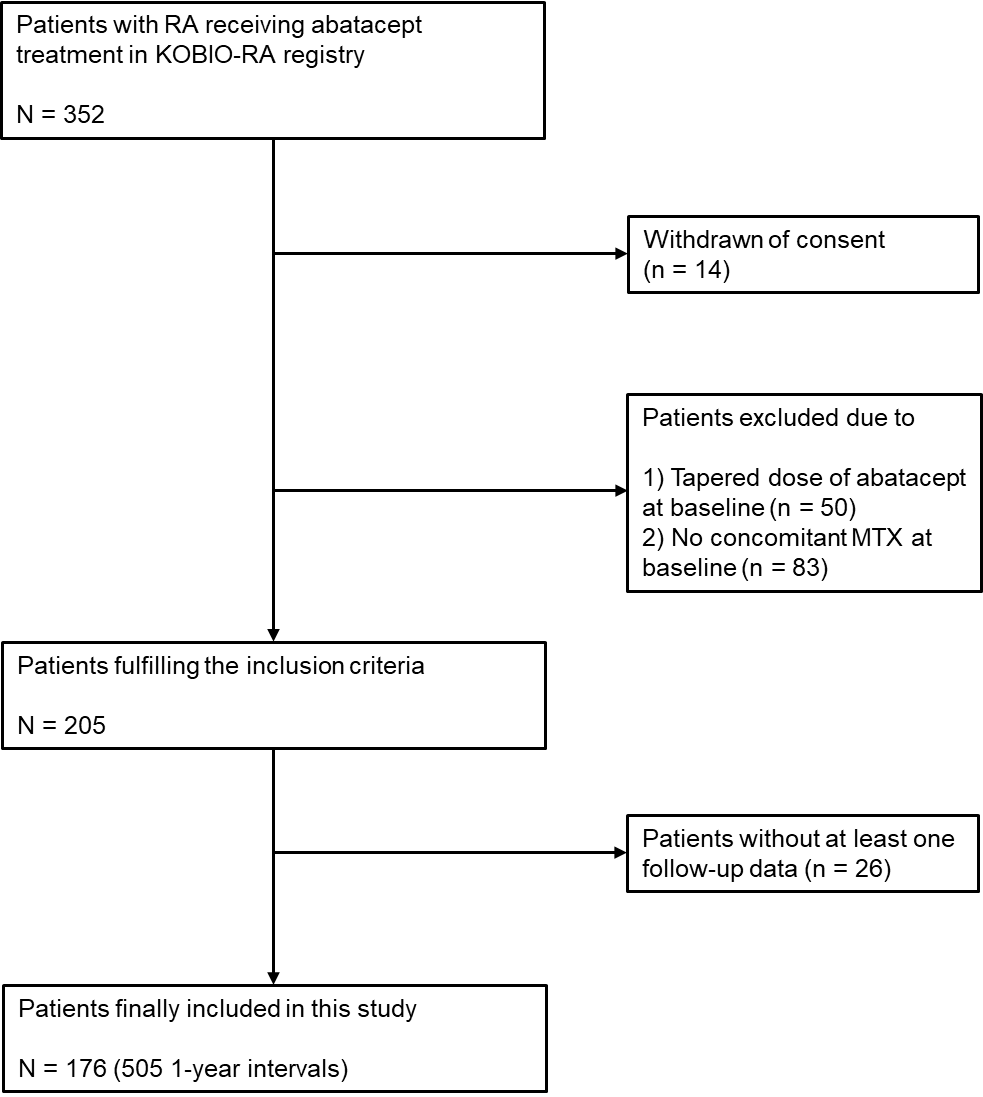


Supplementary Figure 2. Directed acyclic graph of the study


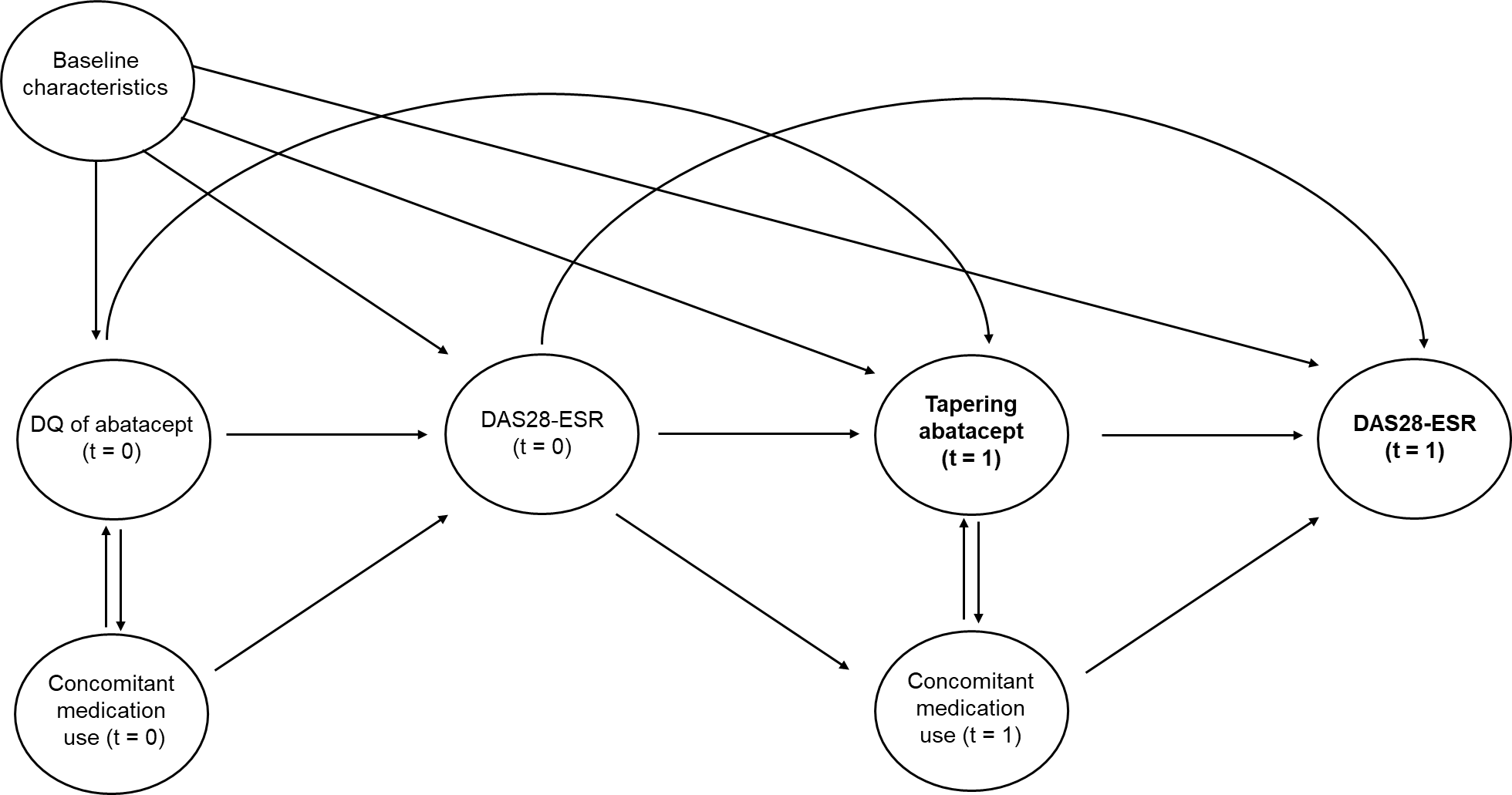


Supplementary Table 1. Baseline characteristics of the study subjects

| Clinical features (n = 179) |  |
| --- | --- |
| Age, years, mean (SD) | 59.3 (12.9) |
| Female sex, n (%) | 150 (83.8) |
| BMI, mean (SD) | 22.4 (3.2) |
| Obesity, n (%) | 43 (24.0) |
| Disease duration, years, mean (SD) | 8.9 (8.3) |
| Smoking, n (%) |  |
| Never | 147 (82.1) |
| Ex- | 16 (8.9) |
| Current | 16 (8.9) |
| Interstitial lung disease, n (%) | 11 (6.1) |
| Comorbidities, n (%) |  |
| Hypertension | 59 (33.0) |
| Diabetes | 21 (11.7) |
| Dyslipidemia | 32 (17.9) |
| Osteoporosis | 62 (34.6) |
| Hypothyroidism | 10 (5.6) |
| Seropositive RA, n (%) | 163 (91.1) |
| bDMARD/tsDMARD-naïve, n (%) | 141 (78.8) |
| Subcutaneous administration, n (%) | 23 (12.8) |
| DAS28-ESR, mean (SD) | 5.66 (1.04) |
| SDAI, mean (SD) | 29.37 (11.13) |
| HAQ, mean (SD) | 1.12 (0.69) |
| Functional impairment, n (%) | 22 (12.3) |
| Dose of concomitant MTX, mg/week, mean (SD) | 12.5 (3.3) |
| Glucocorticoid use, n (%) | 149 (83.2) |
| Dose of glucocorticoid^*^, mg/day, mean (SD) | 4.8 (3.9) |

BMI, body mass index; DAS, disease activity score; DMARD, disease-modifying anti-rheumatic drug; ESR, erythrocyte sedimentation rate; HAQ, Health Assessment Questionnaire; MTX, methotrexate; RA, rheumatoid arthritis; SDAI, simplified disease activity index.

^*^, Based on dose of prednisolone

Supplementary Table 2. Effect of interaction between concomitant MTX and tapering abatacept on disease activity without applying IPTW

|  | Univariable analysis | | Multivariable analysis^a^ | |
| --- | --- | --- | --- | --- |
| Clinical factors | OR (95% CI) | P | OR (95% CI) | P |
| Age at baseline, year | 0.99 (0.98 to 1.01) | 0.420 | ^b^ |  |
| Female sex | 1.11 (0.53 to 2.29) | 0.783 | ^b^ |  |
| Disease duration, year | 0.96 (0.94 to 0.99) | 0.007 | 0.96 (0.93 to 0.99) | 0.005 |
| Smoking |  | 0.064 |  | 0.016 |
| Ex- (reference: never-smoker) | 0.82 (0.32 to 2.07) |  | 0.58 (0.22 to 1.52) |  |
| Current (reference: never-smoker) | 0.41 (0.16 to 1.03) |  | 0.40 (0.19 to 0.84) |  |
| Obesity | 1.10 (0.60 to 2.00) | 0.760 | ^b^ |  |
| Seropositive RA | 1.25 (0.53 to 2.94) | 0.614 | ^b^ |  |
| bDMARD/tsDMARD naïve | 2.37 (1.23 to 4.55) | 0.010 | 2.21 (1.18 to 4.15) | 0.013 |
| Subcutaneous administration | 0.92 (0.46 to 1.83) | 0.805 | ^b^ |  |
| Baseline DAS28-ESR | 0.83 (0.65 to 1.05) | 0.121 | ^b^ |  |
| Baseline HAQ | 0.90 (0.62 to 1.29) | 0.561 | ^b^ |  |
| DAS28-ESR in the previous visit | 0.84 (0.74 to 0.94) | 0.002 | 0.82 (0.71 to 0.94) | 0.005 |
| HAQ in the previous visit | 0.72 (0.52 to 0.99) | 0.045 | 0.83 (0.57 to 1.20) | 0.313 |
| Concomitant MTX | 0.71 (0.38 to 1.32) | 0.278 | ^b^ |  |
| Concomitant MTX * Tapering abatacept |  | 0.017 |  | 0.007 |
| Tapering group with MTX (reference: control group with MTX) | 1.23 (0.83 to 1.83) | 0.303 | 1.22 (0.80 to 1.85) | 0.362 |
| Tapering group without MTX (reference: control group without MTX) | 0.35 (0.14 to 0.89) | 0.028 | 0.28 (0.11 to 0.75) | 0.011 |

DAS, disease activity score; DMARD, disease-modifying anti-rheumatic drug; ESR, erythrocyte sedimentation rate; HAQ, Health Assessment Questionnaire; MTX, methotrexate; OR, odds ratio; RA, rheumatoid arthritis.

^a^, Clinical factors with relevant (*P* < 0.1) association in the univariable analysis were included.

^b^, was not included in the multivariable analysis

Supplementary Table 3. Effect of interaction between concomitant MTX and the extent of abatacept dose tapering on the likelihood of achieving DAS28-remission

|  | Univariable analysis | | Multivariable analysis^a^ | |
| --- | --- | --- | --- | --- |
| Clinical factors | OR (95% CI) | P | OR (95% CI) | P |
| Age at baseline, year | 0.99 (0.98 to 1.01) | 0.420 | ^b^ |  |
| Female sex | 1.11 (0.53 to 2.29) | 0.783 | ^b^ |  |
| Disease duration, year | 0.96 (0.94 to 0.99) | 0.007 | 0.96 (0.93 to 0.99) | 0.001 |
| Smoking |  | 0.064 |  | 0.026 |
| Ex- (reference: never-smoker) | 0.82 (0.32 to 2.07) |  | 0.59 (0.22 to 1.56) |  |
| Current (reference: never-smoker) | 0.41 (0.16 to 1.03) |  | 0.43 (0.20 to 0.92) |  |
| Obesity | 1.10 (0.60 to 2.00) | 0.760 | ^b^ |  |
| Seropositive RA | 1.25 (0.53 to 2.94) | 0.614 | ^b^ |  |
| bDMARD/tsDMARD naïve | 2.37 (1.23 to 4.55) | 0.010 | 2.22 (1.15 to 4.29) | 0.017 |
| Subcutaneous administration | 0.92 (0.46 to 1.83) | 0.805 | ^b^ |  |
| Baseline DAS28-ESR | 0.83 (0.65 to 1.05) | 0.121 | ^b^ |  |
| Baseline HAQ | 0.90 (0.62 to 1.29) | 0.561 | ^b^ |  |
| DAS28-ESR in the previous visit | 0.84 (0.74 to 0.94) | 0.002 | 0.82 (0.72 to 0.95) | 0.006 |
| HAQ in the previous visit | 0.72 (0.52 to 0.99) | 0.045 | 0.83 (0.57 to 1.20) | 0.320 |
| Concomitant MTX | 0.71 (0.38 to 1.32) | 0.278 | ^b^ |  |
| Concomitant MTX * DQ of abatacept ^c^ |  | 0.003 |  | 0.009 |
| DQ (%) with MTX | 1.00 (0.99 to 1.02) | 0.349 | 1.01 (0.99 to 1.02) | 0.336 |
| DQ (%) without MTX | 0.97 (0.95 to 0.99) | 0.004 | 0.96 (0.94 to 0.99) | 0.001 |

DAS, disease activity score; DMARD, disease-modifying anti-rheumatic drug; ESR, erythrocyte sedimentation rate; HAQ, Health Assessment Questionnaire; MTX, methotrexate; OR, odds ratio; RA, rheumatoid arthritis.

^a^, Clinical factors with relevant (*P* < 0.1) association in the univariable analysis were included.

^b^, was not included in the multivariable analysis

^c^, In this analysis, the DQ was multiplied by 100 to show the effect of tapering 1% of the standard abatacept dose.

Supplementary Table 3. Adverse events stratified by concomitant MTX use

|  | Without MTX  (n = 91) | With MTX  (n = 414) | *P* |
| --- | --- | --- | --- |
| Any AEs | 11 (12.1) | 35 (8.5) | 0.275 |
| Leukopenia | 5 (5.5) | 6 (1.4) | 0.017 |
| Infusion/injection site reaction | 1 (1.1) | 3 (0.7) | 0.715 |
| Tuberculosis infection | 0 (0.0) | 0 (0.0) | NA |
| NTM infection | 0 (0.0) | 1 (0.2) | 0.639 |
| Bacterial infection | 1 (1.1) | 5 (1.2) | 0.931 |
| Fungal infection | 0 (0.0) | 1 (0.2) | 0.639 |
| Herpes zoster infection | 0 (0.0) | 7 (1.7) | 0.212 |
| LFT abnormality | 1 (1.1) | 1 (0.2) | 0.238 |
| Skin rash | 0 (0.0) | 7 (1.7) | 0.212 |
| Malignancy | 3 (3.3) | 2 (0.5) | 0.014 |
| Interstitial lung disease | 1 (1.1) | 6 (1.4) | 0.796 |
| SAEs | 4 (4.4) | 7 (1.7) | 0.110 |
| Bacterial infection | 1 (1.1) | 3 (0.7) | 0.715 |
| Fungal infection | 0 (0.0) | 1 (0.2) | 0.639 |
| Malignancy | 3 (3.3) | 2 (0.5) | 0.014 |
| Interstitial lung disease | 0 (0.0) | 2 (0.5) | 0.506 |

AE, adverse event; LFT, liver function test; NTM, Nontuberculous mycobacteria; NA, not applicable; SAE, serious adverse event.
